# Animal models of pediatric abusive head trauma

**DOI:** 10.1007/s00381-022-05577-6

**Published:** 2022-06-10

**Authors:** John W. Finnie, Peter C. Blumbergs

**Affiliations:** 1 Discipline of Anatomy and Pathology, Adelaide Medical School, Adelaide, Australia; 2 Office of the Deputy Vice-Chancellor (Research), University of Adelaide, Adelaide, South Australia 5006 Australia

**Keywords:** Abusive head trauma, Animal models, Neuropathologic changes, Pathogenetic mechanisms

## Abstract

**Background:**

Abusive head trauma (AHT), previously known as the shaken baby syndrome, is a severe and potentially fatal form of traumatic brain injury in infant children who have been shaken, and sometimes also sustained an additional head impact. The clinical and autopsy findings in AHT are not pathognomonic and, due to frequent obfuscation by perpetrators, the circumstances surrounding the alleged abuse are often unclear. The concept has evolved that the finding of the combination of subdural hemorrhage, brain injury, and retinal hemorrhages (“the triad”) is the result of shaking of an infant (“shaken baby syndrome”) and has led to the ongoing controversy whether shaking alone is able to generate sufficient force to produce these lesions.

**Objective:**

In an attempt to investigate whether shaking can engender this lesion triad, animal models have been developed in laboratory rodents and domestic animal species. This review assesses the utility of these animal models to reliably reproduce human AHT pathology and evaluate the effects of shaking on the immature brain.

**Results:**

Due largely to irreconcilable anatomic species differences between these animal brains and human infants, and a lack of resemblance of the experimental head shaking induced by mechanical devices to real-world human neurotrauma, no animal model has been able to reliably reproduce the full range of neuropathologic AHT changes.

**Conclusion:**

Some animal models can simulate specific brain and ophthalmic lesions found in human AHT cases and provide useful information on their pathogenesis. Moreover, one animal model demonstrated that shaking of a freely mobile head, without an additional head impact, could be lethal, and produce significant brain pathology.

## Introduction

Abusive head trauma (AHT) is the currently accepted designation for intentionally inflicted head injury in young children as it does not attribute the head injuries to any specific pathogenetic mechanism [1]. This neurologic condition has also been termed “pediatric non-accidental head injury,” “the shaken baby syndrome,” “battered baby syndrome,” “whiplash shaking injury” [2, 3], and “the shaken impact syndrome” [4–6].

The spectrum of neuropathologic changes in AHT includes acute and chronic subdural hemorrhage (SDH), cerebral swelling, intracerebral hemorrhage, contusional tears, hypoxic-ischemic damage, and traumatic and ischemic axonal injury (AI). The pathophysiology and biomechanics producing these lesions, singly or in various combinations, vary from case to case [2, 3].

The pattern of acute encephalopathy found in AHT is global and ischemic, neuronal injury being widely distributed, and characterized by cytoplasmic shrinkage and eosinophilia (red neurons). Hypoxia-induced diffuse cerebral swelling is the usual cause of death [7, 8]. Diffuse axonal injury (DAI) is infrequent in this age group of children [7, 8], but vascular AI due to raised intracranial pressure is a frequent finding. Often, there is a mix of ischemic and traumatic patterns of AI. The craniocervical (cervicomedullary) junction appears to be particularly vulnerable to traumatic injury in infants less than 3 months of age, and the ischemic-hypoxic AI found at this site in AHT has been proposed as the structural basis for children presenting with apnea and/or dyspnea. This AI could lead to cardiorespiratory arrest and subsequent global cerebral ischemia and swelling [7–9]. Unilateral brain lesions, however, are less convincingly explained by this mechanism [3].

Acute SDH is very often present in suspected AHT cases and, although it is not specific for this condition, prompts a consideration of abuse, in the absence of any other possible etiology [2, 3]. SDH in AHT cases is characterized by a thin layer of blood, which is usually bilaterally distributed. Shearing forces generated by acceleration/deceleration of the head are the most likely cause of ruptured bridging veins resulting in SDH [2], although hypoxic endothelial damage in these immature veins, with increased vascular permeability, has also been proposed as a putative injury mechanism [10].

Retinal hemorrhage (RH) is more common and severe in AHT than accidental injury in infants, but it is still non-specific, as there are many other potential causes [2, 3]. In AHT, these hemorrhages are generally extensive and involve all retinal layers. They are believed to be caused by rotational acceleration/deceleration forces [2, 3], although it has been speculated that RH could be due to hypoxic vascular damage [10].

## Animal models of AHT

Published studies of AHT have often been criticized for their case selection bias, and systematic reviews have concluded that there is insufficient scientific, evidence-based research to assess the diagnostic accuracy of the “triad” in positively diagnosing AHT [11–16] and controversy exists whether the triad can be produced by shaking alone. Moreover, a diagnosis of AHT is often hampered because a convincing history of the circumstances surrounding a suspected case of AHT is lacking, due to obfuscation by the alleged perpetrator, and there is no reliable witness. The absence of a contact injury also does not necessarily mean that there was no head impact.

Accordingly, a major aim of animal modeling should be to better understand the pathogenesis and biomechanics of the pathologic changes found in AHT.

There are a number of features of pediatric brains that must be considered when developing models of AHT, and these attributes explain why attempting to extrapolate data from adult traumatic brain injury (TBI) studies to the pediatric population is often problematic. Incomplete myelination of infant brains may account for the differing response to a traumatic insult from that of an adult, especially as lipids such as myelin have a low shear value [17, 18]. The increased vulnerability to shear injury caused by incomplete myelination, and astrocytic immaturity, also seems to favor diffuse brain damage [19].The infant bony cranial vault is soft and pliable, with unfused sutures, fontanelles are open, bone and dura are very vascular, the dura is not firmly attached to the inner table of the skull, the relatively large subarachnoid space has abundant blood vessels, and smooth bony buttresses at the base of the skull offer little resistance to brain movement [19]. The higher water content of the immature brain makes it less compressible, but also more susceptible to elevated ICP, although this is offset to some extent by patent fontanelles and the potential for suture diastasis. An infant brain can sustain significantly greater deformation than that of older children, with greater forces generally being required to deform the pediatric brain. While the sutures in an infant skull can deform over 100% before rupturing, an adult skull shows an 11-fold greater resistance to mechanical force, for the stiffness of the cranial bones increases with age. However, while a more deformable skull may lessen diffuse brain injuries caused by angular acceleration, it may increase local injuries beneath the impact site [19–21].

The timing of the growth spurt, the transient period of rapid brain growth, must also be taken into account when extrapolating results from one species to another. It is important to compare the brains of different species at comparable developmental stages rather than age relative to birth [22]. According to this brain growth spurt, mammalian species can be categorized as predominantly pre-natal (for example sheep), perinatal (humans, pigs), and postnatal (rats, mice) brain developers. While the brain growth spurt varies considerably between species, it is, nevertheless, recognized as being especially vulnerable to developmental damage from a wide range of insults [22].

While there are large gaps in our knowledge of the pathophysiologic and biomechanical mechanisms involved in the development of AHT brain lesions, and these studies cannot be ethically conducted in humans, very few animal models have been devised to study the pathogenesis of AHT.

The principal focus of this review is to assess the utility of animal models which have been developed to study the neuropathologic changes occurring in AHT. An evaluation of the biomechanical models of AHT is beyond the scope of this review, but they are invaluable to analyze head movements following inflicted injury, or differential movements within the brain or between brain and skull. Shaking and head impact studies require very different biomechanical modeling, the former being a relatively low intensity, long duration event, while the latter involves high intensity, short duration forces. It has often been difficult to compare results between different biomechanical models, due to diverse models types and injury mechanisms, and experimental findings have sometimes been contradictory [23].

Similar to biomechanical studies, a comparison of the neuropathologic changes found between different animal models of AHT is difficult because of the range of mechanical devices developed to produce head movements in a variety of directions, and in different animal species. Moreover, the head is usually constrained in these devices to enhance the reproducibility of results, rather than being freely mobile, and a variety of histologic techniques have been used to detect brain lesions. Nevertheless, an attempt was made (Table 1) in this review to assess the reliability of these models in reproducing neuropathologic changes relevant to human AHT, in terms of both their consistency of occurrence, sufficient degree of severity, and wide neuroanatomic distribution. The lesions evaluated were ischemic-hypoxic neuronal injury (presenting as neuronal cytoplasmic shrinkage and eosinophilia), multifocal AI in diverse white matter tracts, blood–brain barrier (BBB) breakdown and resulting diffuse vasogenic edema, and SDH/SAH and RH. If a lesion was not described in a given study, it was assumed that it was not evaluated.

**Table 1:** Neuropathologic changes in animal models of AHT

Research group	Animal species/age	Brain injury mechanism	Head movement and direction	Neuropathologic changes*				
				Ischemic-hypoxic neuronal damage	Multifocal white matter axonal injury	BBB disruption/diffuse vasogenic edema	Subdural/subarachnoid hemorrhage	Retinal hemorrhage
Smith et al. (1998) [31]	6-day-old rats	Mechanical shaking with hypoxia	Horizontal; constrained	ND	ND	ND	+	ND
Bonnier et al. (2002, 2004) [28, 29]	8-day-old mice	Rotational mechanical shaking	Horizontal; unrestrained	ND	+	ND	-	+
Wang et al. (2018) [27]	12-day-old mice	Mechanical shaking	Sagittal; constrained	ND	+	+	+	-
Raghupathi and Margulies (2002) [30]	3–5-day-old piglets	Rotational mechanical shaking	Axial plane; constrained	ND	+	ND	+	ND
Eucker et al. (2011) [24]	3–5-day old piglets	Rotational mechanical shaking	Horizontal/sagittal/coronal planes; constrained	+	+	ND	+	ND
Finnie et al. (2010, 2012) [25, 26]	7–10-day-old lambs	Naturalistic manual shaking	Horizontal/lateral/rotational; freely mobile	-	+	+	+	+
Coats et al. (2010) [37]	3–5-day-old piglets	Rotational mechanical shaking	Axial/sagittal/coronal; constrained	N/A	N/A	N/A	N/A	+

* + lesion produced with consistency/sufficient severity and wide neuroanatomic distribution, - not found, *ND* not described, *N/A* not applicable

It is evident from this analysis that no animal model developed to date has reliably reproduced the full range of neuropathologic changes found in abused human infants, although some models could be potentially useful to study the pathogenesis of specific lesions.

**Fig. 1:**
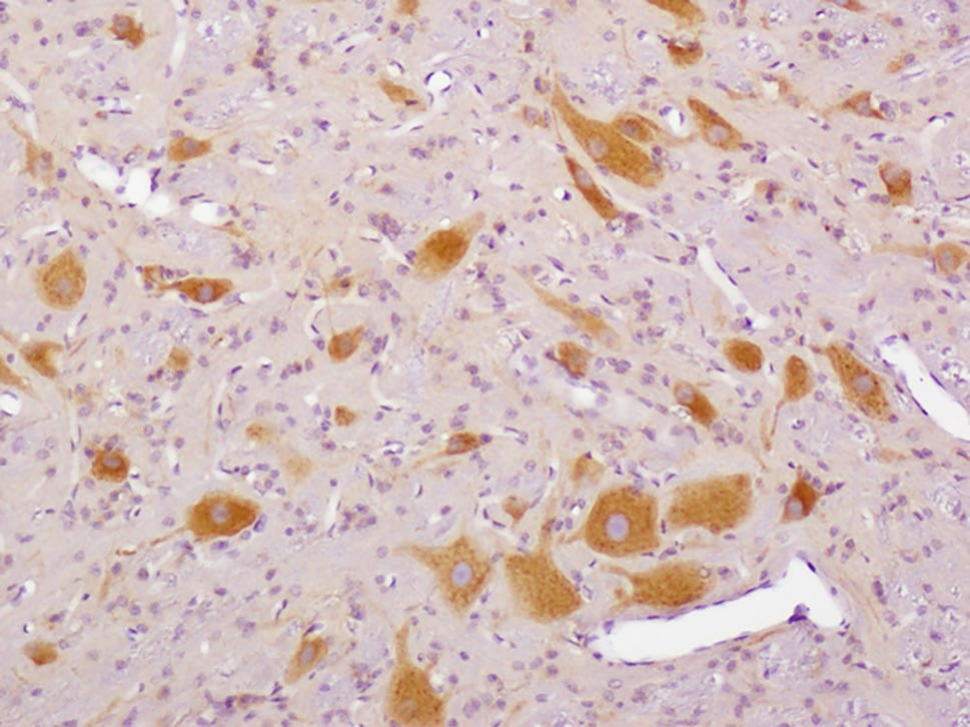
Marked neuronal APP immunopositivity in the cervical spinal cord (Fig. 1) and retinal ganglion cell layer (Fig. 2). Injured ganglion cell axons (Fig. 2) are also APP-immunopositive (arrows). (Original magnification ×10 (Fig. 1) and ×40 (Fig. 2))

**Fig. 2:**
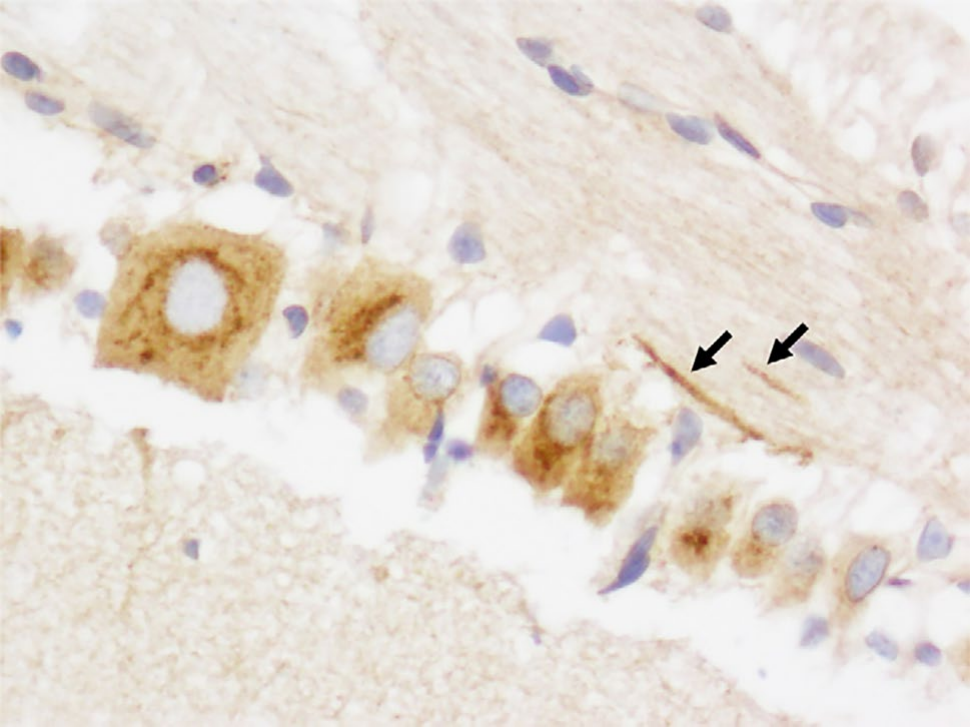
Marked neuronal APP immunopositivity in the cervical spinal cord (Fig. 1) and retinal ganglion cell layer (Fig. 2). Injured ganglion cell axons (Fig. 2) are also APP-immunopositive (arrows). (Original magnification ×10 (Fig. 1) and ×40 (Fig. 2))

**Fig. 3:**
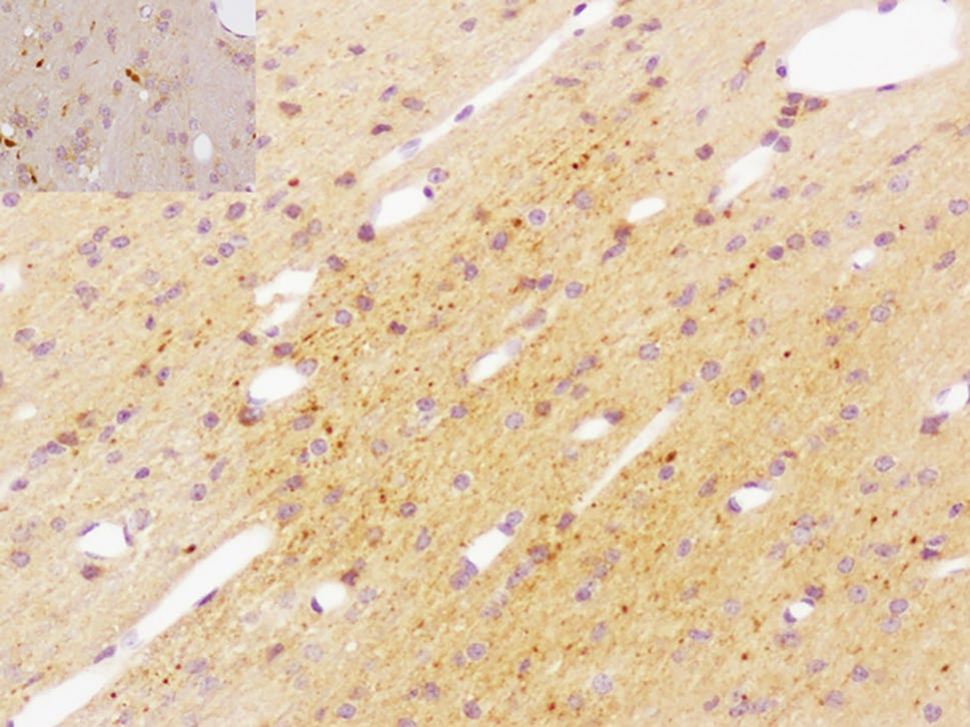
Numerous APP-immunopositive damaged axons in the hemispheric white matter (higher power in the inset). (Original magnification ×10 (×40 inset))

**Fig. 4:**
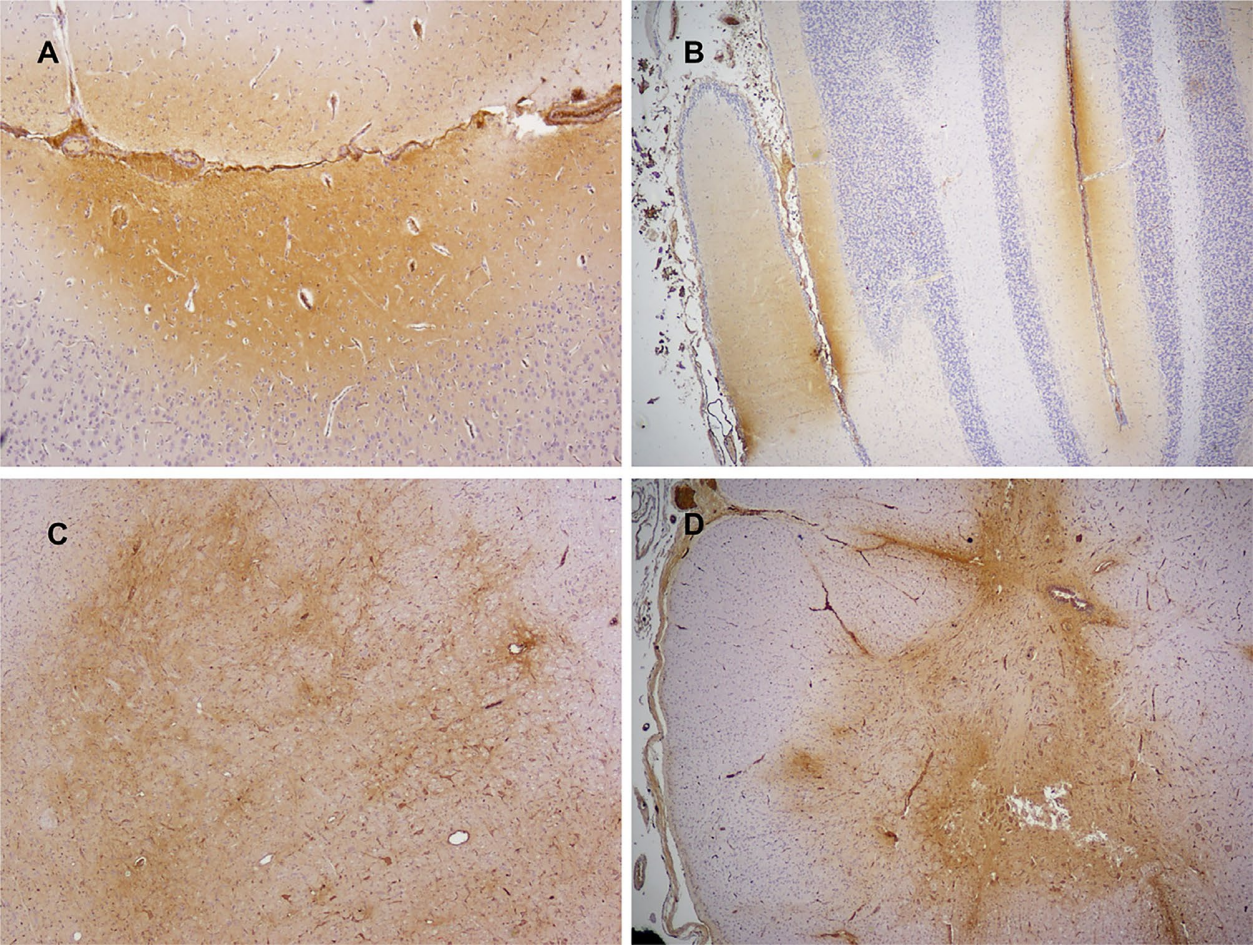
Widespread BBB breakdown in the cerebral cortex (**A**), cerebellar folia (**B**), brainstem (**C**), and cervical spinal cord (**D**) using immunohistochemical labeling of extravasated albumin as a surrogate marker of increased vascular permeability. (Original magnification ×4)

The only model to reliably produce widespread neuronal ischemic-hypoxic injury was that developed by the University of Pennsylvania, in which 3–5-day-old piglets sustained a head rotation in different directions [24]. However, widely distributed neuronal APP immunopositivity in the brain (Fig. 1), and retinal ganglion cells (Fig. 2), was found in a lamb model of AHT [25, 26]. This neuronal response was considered to represent a non-specific, acute stress response to manual shaking. Using a silver impregnation technique, progressive neocortical neuronal degeneration was also found in shaken infant mice [27]. Multifocal AI in diverse white matter tracts was observed in a few of these animal models, being detected (generally with amyloid precursor protein (APP) immunohistochemistry, APP being the most sensitive, early marker of AI [2]) in 8- and 12-day-old shaken mice [27–29], after mechanical head rotation in 3–5-day-old piglets [24, 30], and in manually shaken, 7–10-day-old lambs [25, 26]. In the lambs, AI was particularly severe in the hemispheric white matter (Fig. 3) but also widely distributed in the brainstem and at the craniocervical junction, the latter the site of maximal loading during shaking. It was posited that AI at the craniocervical junction may have caused apnea and cardiorespiratory arrest in a subset of lambs that died. Evidence of BBB disruption, with subsequent diffuse vasogenic edema, was observed in 12-day-old mice subjected to mechanical, sagittally directed, extension-flexion of the head [27] and manually shaken lambs [25, 26], the latter using immunohistochemistry for plasma albumin as a surrogate marker of increased vascular permeability (Fig. 4). In a mouse model [28, 29], parenchymal damage was instead restricted to the periventricular white matter, resulting in hemorrhage and, eventually, cystic change, although AI was more widespread. SDH/SAH was found in some animal models [24–27, 30, 31], but RH was uncommon. SDH was modeled in 3-week-old piglets [32], but exogenous blood was infused into the subdural space via craniotomy, rather than SDH being produced by mechanically induced head movements. In a manually shaken lamb model [33], striking expression of the immediate early gene, *c-fos*, was found in meningothelial cells in the cranial cervical spinal cord, but also in hemispheric, cerebellar, and brainstem meninges, and vascular endothelial cells in meninges and hemispheric white matter. This reaction was hypothesized to be due to mechanical stress induced by shaking, with differential movement of different craniospinal components.

The University of Pennsylvania porcine model was used to study brain lesions produced by mechanical head shaking in different directions, and of varying applied frequency. Piglets were selected because they resemble the human postnatal brain development sequence, and have similar cerebrovascular development, and responses such as cerebral blood flow and brain electrical activity [21]. It was concluded from these porcine studies that, overall, the severity of neuropathologic changes, particularly AI, was determined by the number of traumatic shaking insults sustained, the time interval between these insults, and the rotational direction of the head movement induced by the impulsive force. Traumatic AI in 3–5-day-old piglets was shown to be greater after repeated than a single head rotation, implying a cumulative effect of this traumatic insult [34, 35]. AI was also greater when the rotations were 24 h rather than 7 days apart [35, 36]. Cyclic, low velocity head rotations in these piglets produced more AI than a single rotation of the same magnitude [36]. These piglets were also used in the same mechanical device to study brain lesions produced by head rotation in different directions [24]. Head movements in the sagittal and horizontal, but not coronal, directions frequently produced ischemic-hypoxic neuronal damage, multifocal AI, and SDH.

In order to study the pathogenesis of the ocular hemorrhage found in AHT, 3–5-day-old piglets were used in the University of Pennsylvania mechanical device [37]. After head movement in different directions, three-quarters of the animals showed RH 6 h post-injury, and 70% of these hemorrhages were located in a region of strong vitreoretinal attachment. More hemorrhages were produced by axial than sagittal or coronal rotations. These findings supported the notion that acceleration/deceleration forces cause abnormal traction of the retina by the attached vitreous, thereby damaging retinal blood vessels and causing hemorrhage. In terms of translation of these ophthalmic findings to human AHT, the porcine eye has vitreoretinal attachments across the entire retina, resembling that of humans, and does not have a tapetum; it does not, however, possess a fovea or macula. The ovine eye is also similar to humans with respect to both vitreoretinal adhesions and a holangiotic retinal vasculature, in which the entire neuroretina is supplied by the intraretinal circulation. Using pre-term lambs, it was found that their immature vasculature was more vulnerable to injury than that of adults because the vessel lengths were short, and more highly branched, thereby rendering them more likely than mature retinal blood vessels to sustain greater stress and strain during traumatic loading [38].

## Discussion

The experimental animal models, in both laboratory rodents and domestic animal species, developed to date were unable to reliably replicate the full spectrum of neuropathologic changes found in human infant AHT. This was due largely to irreconcilable neuroanatomic species differences, and the fact that the head was usually constrained, rather than freely mobile, in the mechanical devices used to inflict shaking injuries in different directions. With respect to the latter, it is unfortunately a common attribute of animal models of TBI that the more controlled and reproducible the mechanical input, the less the model resembles real-world human neurotrauma, and the more problematic the translatability of the experimental results to human patients.

In most laboratory rodent models, and the University of Pennsylvania porcine model, the head was limited to movement only in certain specified directions, which does not simulate the likely head motions that occur when a human infant is maliciously shaken. It was only in the shaken lamb model [25, 26] that a large head was freely mobile, these animals being manually grasped under the axilla and shaken with sufficient force to snap the head back and forth onto the chest, similar to the head motion believed to occur in human AHT. Biomechanical studies established that, in addition to acceleration/deceleration of the unrestrained lamb head in the axial plane, there was also considerable lateral and rotational head movement [39].

One of the most contentious issues in AHT, which animal models are yet to resolve, is whether shaking alone is sufficient to produce significant brain damage, or whether an additional head impact is required. It was found that the angular acceleration shaking forces engendered in a surrogate model of a 1-month-old human infant [4] were well below, by a factor of 50, those believed to be required to cause concussion, SDH, and DAI in non-human primates [40]. However, the naturalistic lamb manual shaking model [25, 26] demonstrated that shaking of a freely mobile head could be lethal, without a head impact being needed. In this model, lambs were manually shaken 10 times, of 30-s duration, over a 30-min period, and killed 6 h post-injury. A subset of lower body weight lambs died before the designated 6-h survival period.

Since human TBI is composed of a complex constellation of primary and secondary injury cascades, of varying severity and regional distribution [2], it is very difficult for a single animal model to replicate the complete spectrum of neuropathologic changes found in these traumatized brains. However, animal models can address specific types of mechanical input and resulting lesions tend to be more homogeneous and amenable to analysis [21]. The heterogeneous nature of human TBI also explains why translation of experimental results from animal models to humans can often be unrewarding.

In order to understand why animal models have often proved to be unsatisfactory for the study of AHT, it is useful to examine some of the important differences between the brains of laboratory rodents, and domestic animal species, and those of humans. Rodents, such as mice and rats, have small, lissencephalic brains with scant white matter, the latter making characterization of AI difficult. Rodent brains are devoid of convolutions, the presence of gyri affecting the movement of the brain within the skull. Moreover, significantly more brain deformation occurs after head impact in a brain with gyri compared to one without [41, 42]. Since shearing forces and inertial loading are related to brain mass, small rodent brains can tolerate much greater acceleration/deceleration forces than animals with larger, gyrencephalic brains, necessitating that very high rotational forces need to be applied to the rodent brain to simulate loads experienced in human AHT [42]. The margin between a fatal and non-fatal head impact in rodents is also very slender [42]. Furthermore, a smooth brain surface can tolerate deformation more readily than brains with well-developed gyri [3]. In addition to differences in the distribution of gray and white matter between the rodent brain and human infants, rodents also have limited fidelity to human proteomic and genomic responses, and different injury time courses. Moreover, the peak growth spurt of the rodent brain occurs before birth, while that of humans and pigs occurs around birth [3, 42]. Changes in myelination and water content of the pig brain during development also resemble those in humans [22].

While domestic animal species, such as sheep and pigs, have greater anatomic and physiologic similarity to humans, including having relatively large, gyrencephalic brains with well-developed gyral convolutions, the orientation of the neuraxis in these species is very different from that of humans. The almost linear axis in quadrupeds may also impede rotational shearing after TBI and render the animal less vulnerable to concussion. In addition to gyrencephalic brains, lambs also have weak neck muscles, resembling a human infant, which is an important anatomic feature when replicating human AHT. Relative to its body size, the human infant head is significantly larger than that of an adult, and the weak cervical paraspinal muscles mean that an infant has poorer control over movement of this disproportionately large head. Infants are, therefore, at increased risk of rotational and rapid acceleration/deceleration injuries during shaking, due to significant differential movement of the immature brain with respect to the skull [3].

In the setting of an immature human brain, there are some special features of its response to traumatic injury that need to be considered when analyzing the experimental findings in animal models of AHT. While the immature brain seems to be more resistant to ischemia-hypoxia, this lesion is, nevertheless, a major contributor to secondary brain damage occurring after TBI. The immature BBB is also more vulnerable to ischemic injury, resulting in diffuse rapid swelling of the brain, often more congestive than edematous. Mass lesions developing from brain hemorrhage appear to cause fewer unfavorable clinical neurologic outcomes in children than adults [43], and there is a progressive shift from largely apoptotic neuronal death to necrosis with increasing developmental age [44, 45]. During the different stages of brain maturation, there are also changing patterns of autoregulation of cerebral blood flow, metabolic enzyme activity, oxidative stress, neurotransmission, mitochondrial function, and neuroplasticity, factors which can be beneficial or detrimental [12].

In conclusion, it is unlikely that any animal model will be able to precisely replicate the complete range of brain and ocular lesions used to support a diagnosis of AHT in human infants, and the model selected will probably be one that is most appropriate to the specific lesion under investigation. The profound differences between immature laboratory rodent, and to a lesser extent domestic animal, brains, and those of human infants will make inter-species translation of experimental results often problematic, especially if the animal heads are constrained during shaking, rather than being freely mobile. Notwithstanding these difficulties, animal models have greatly improved our understanding of the pathogenesis of adult human TBI, and they will hopefully be further improved to generate findings that will lead to a more reliable neuropathologic diagnosis of AHT.
